# Development of in-house fully residual deep convolutional neural network-based segmentation software for the male pelvic CT

**DOI:** 10.1186/s13014-021-01867-6

**Published:** 2021-07-22

**Authors:** Hideaki Hirashima, Mitsuhiro Nakamura, Pascal Baillehache, Yusuke Fujimoto, Shota Nakagawa, Yusuke Saruya, Tatsumasa Kabasawa, Takashi Mizowaki

**Affiliations:** 1 Department of Radiation Oncology and Image-Applied Therapy, Graduate School of Medicine, Kyoto University, 54 Kawahara-cho, Shogoin, Sakyo-ku, Kyoto, 606-8507 Japan; 2 Division of Medical Physics, Department of Information Technology and Medical Engineering, Human Health Sciences, Graduate School of Medicine, Kyoto University, 53 Kawahara-cho, Shogoin, Sakyo-ku, Kyoto, 606-8507 Japan; 3 Rist, Inc., Impact HUB Tokyo, 2-11-3 Meguro, Meguro-ku, Tokyo, 153-0063 Japan

**Keywords:** Computed tomography, Fully residual deep convolutional neural network, Segmentation accuracy, Male pelvic region

## Abstract

**Background:**

This study aimed to (1) develop a fully residual deep convolutional neural network (CNN)-based segmentation software for computed tomography image segmentation of the male pelvic region and (2) demonstrate its efficiency in the male pelvic region.

**Methods:**

A total of 470 prostate cancer patients who had undergone intensity-modulated radiotherapy or volumetric-modulated arc therapy were enrolled. Our model was based on FusionNet, a fully residual deep CNN developed to semantically segment biological images. To develop the CNN-based segmentation software, 450 patients were randomly selected and separated into the training, validation and testing groups (270, 90, and 90 patients, respectively). In Experiment 1, to determine the optimal model, we first assessed the segmentation accuracy according to the size of the training dataset (90, 180, and 270 patients). In Experiment 2, the effect of varying the number of training labels on segmentation accuracy was evaluated. After determining the optimal model, in Experiment 3, the developed software was used on the remaining 20 datasets to assess the segmentation accuracy. The volumetric dice similarity coefficient (DSC) and the 95th-percentile Hausdorff distance (95%HD) were calculated to evaluate the segmentation accuracy for each organ in Experiment 3.

**Results:**

In Experiment 1, the median DSC for the prostate were 0.61 for dataset 1 (90 patients), 0.86 for dataset 2 (180 patients), and 0.86 for dataset 3 (270 patients), respectively. The median DSCs for all the organs increased significantly when the number of training cases increased from 90 to 180 but did not improve upon further increase from 180 to 270. The number of labels applied during training had a little effect on the DSCs in Experiment 2. The optimal model was built by 270 patients and four organs. In Experiment 3, the median of the DSC and the 95%HD values were 0.82 and 3.23 mm for prostate; 0.71 and 3.82 mm for seminal vesicles; 0.89 and 2.65 mm for the rectum; 0.95 and 4.18 mm for the bladder, respectively.

**Conclusions:**

We have developed a CNN-based segmentation software for the male pelvic region and demonstrated that the CNN-based segmentation software is efficient for the male pelvic region.

**Supplementary Information:**

The online version contains supplementary material available at 10.1186/s13014-021-01867-6.

## Introduction

High-precision radiotherapy, including intensity-modulated radiotherapy (IMRT) and volumetric-modulated radiotherapy (VMAT) comprise five steps: computed tomography (CT) simulation, segmentation, treatment planning, patient-specific quality assurance, and treatment. Among these, segmentation is time-consuming and associated with inter-observer variations [1]. Auto-segmentation methods are preferred for workload alleviation. Atlas-based segmentation methods have been used in clinical practice [2–4]. An atlas is a library of organs-at-risk derived by manual segmentation, and the data are extrapolated to new patients via image registration [2]. This reduces the physician segmentation time by 30–40% as well as inter-observer variation [3]; however, because the method is sensitive to atlas selection and strongly dependent on registration accuracy, it is difficult to generalize the data [4]. Therefore, a next-generation auto-segmentation method is required.

Recently, deep learning methods have been used to identify objects in images [5, 6]. Deep learning auto-segmentation algorithms have rapidly become state-of-the-art in terms of medical image segmentation [6]. Convolutional neural networks (CNNs) are learning methods featuring multiple levels of representation. Units in a convolutional layer are organized into feature maps, within which each unit is connected to local patches of the feature maps of the previous layer via a set of weights. Auto-segmentation using CNNs featuring deep architectures improved segmentation accuracy and decreased the segmentation time compared to the atlas-based method [7, 8].

Generally, high-quality performance is obtained using a larger number of data for the CNN-based model. In the male pelvic region, the auto-segmentation accuracy was slightly improved when a large dataset was used to create a model to perform the auto-segmentation via the CNN [9–14]; nevertheless, the effect of the size of the dataset on the segmentation accuracy has not been explored. In addition, practically, multi-labeling of medical images prior to segmentation is a major problem. Real images exhibit many individual anatomical intricacies caused by variations in organ shapes and sizes. Moreover, organs evident on the CT images of the male pelvic region contrast poorly, and the surrounding boundaries of the prostate, seminal vesicles, rectum, and bladder may not be clearly visible. Multi-labeling has been used for segmentation in many contexts; therefore, a unique network has been developed to solve the problem [15–17]. We hypothesized that annotation differences such as changing the number of labels, would affect the segmentation accuracy. Whether varying the number of labels for training improves segmentation accuracy has not yet been investigated.

Furthermore, auto-segmentation has great real-world clinical potential with the possibility of reducing time consumption [18]. Despite the number of published studies in this area, it is difficult to generalize these outcomes because it can only be used with dedicated treatment planning support systems [19–25]. Therefore, in this study, we develop and evaluate the accuracy of a software that can be used on the commercial radiation treatment planning system (RTPS).

This study aimed to: (1) develop a fully residual deep CNN-based segmentation software for the male pelvic region and (2) demonstrate its efficiency in prostate cancer patients.

## Materials and methods

### Patient data

We enrolled 470 prostate cancer patients who had undergone IMRT or VMAT in the prone position at our institution between July 2007 and August 2015 in our study. CT images were acquired using a matrix of 512 × 512 and a 2.5 mm slice thickness (voxel size, 0.97 × 0.97 × 2.5 mm) on the LightSpeed RT platform (GE Healthcare, Little Chalfont, UK). Region of interest (ROIs) of the prostate, seminal vesicles, rectum, and bladder were manually delineated by experienced radiation oncologists and medical physicists. The rectal ROI ran from 15 mm below the apex of the prostate to 15 mm above the tips of the seminal vesicles. Patients who underwent femoral head replacement were not included. The study was approved by our institutional review board and adhered to all relevant ethical tenets of the Helsinki Declaration (R1499).

### Model architecture and implementation

Our model was based on FusionNet [26], a fully residual deep CNN developed to semantically segment biological images. The FusionNet architecture features many ReLU convolution layers, including down-sampling and up-sampling layers. In the model, a 512 × 512 input is gradually transformed into a 32 × 32 representation and finally expanded to a probability map of the same size as the input. All the trainings and predictions were performed using the Intel Core Xeon CPU, single NVIDIA Tesla V100 GPU, and 244 GB RAM in the Python 3.6 environment. Our model was written in Keras featuring a TensorFlow backbone. During the training, we determined all model hyper-parameters experimentally. We used unbalanced weights for all the labels and set them by referencing the total inverse area of each label. The model was trained using a mini-batch approach (size: 28) and the Adam algorithm. The learning rate was set to 0.001 to allow optimization.

### Experiments

**Fig. 1:**
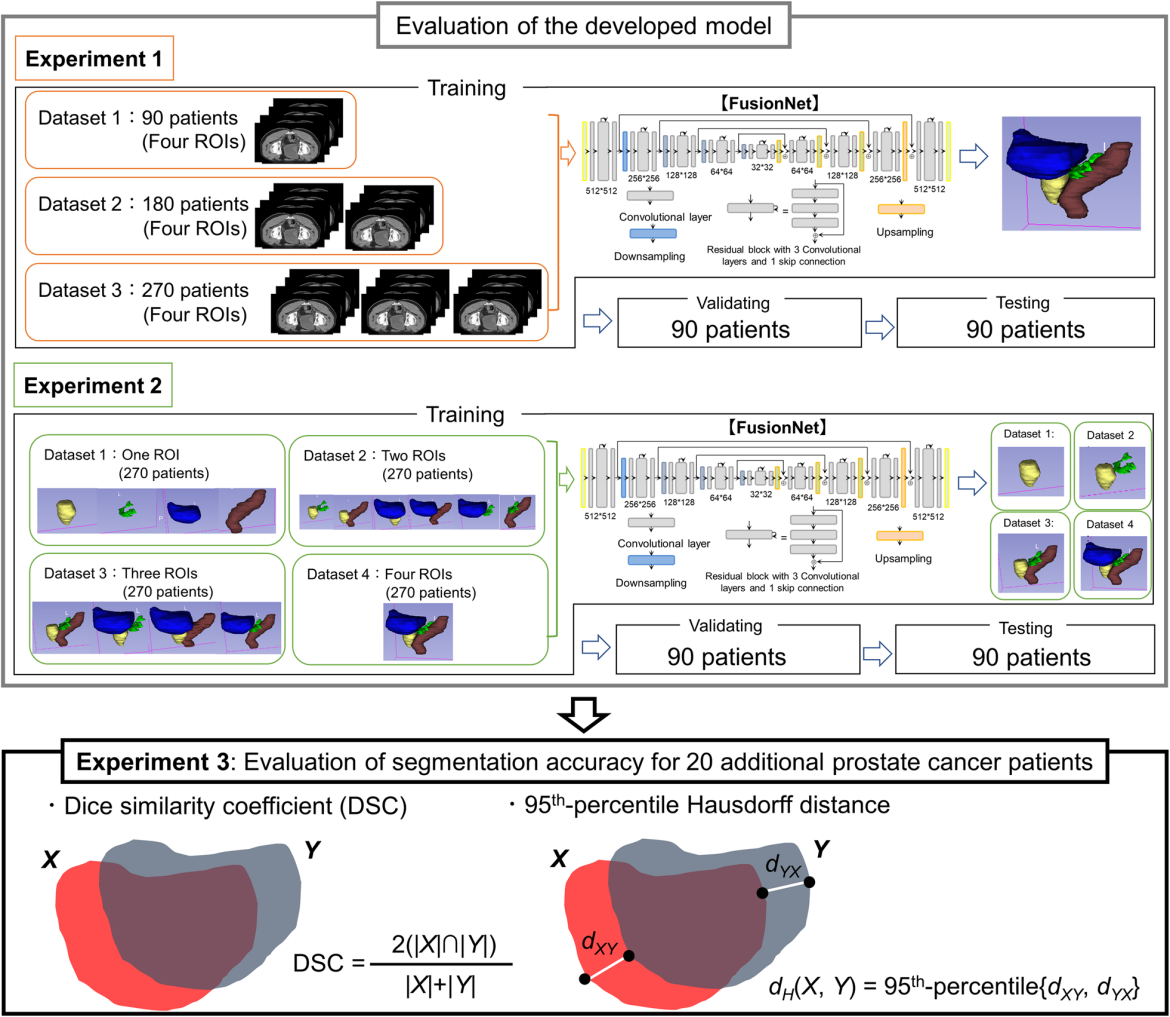
Overall strategy of the study. Experiments 1 and 2 evaluated the effects of dataset size and label number, respectively, on segmentation accuracy to determine the optimal model. Thereafter, Experiment 3 was performed to evaluate the segmentation accuracy in 20 additional prostate cancer patients

The overall strategy in this study is shown in Fig. 1. To determine an optimal segmentation model, we first calculated the volumetric dice similarity coefficient (DSC) when the training dataset changed and then explored various learning strategies in Experiments 1 and 2, respectively. In Experiments 1 and 2, the 450 patients and the corresponding structural images were randomly separated into the training (270 patients; 60%), validation (90 patients; 20%), and testing (90 patients; 20%) datasets. To build robust models using a limited dataset, we randomly augmented all images via rotation (± 15°) and shearing (± 0.1 radians) during the training. The model was validated using the DSC of the validation dataset and set the upper limit of the training iteration to 100 epochs. The details of Experiments 1 and 2 are described in the subsequent sections.

Based on the results obtained in Experiments 1 and 2, we developed a CNN-based segmentation software. In Experiment 3, the segmentation accuracy was then evaluated in 20 additional prostate cancer patients using the developed software.

### Experiment 1: segmentation accuracy with datasets of different sizes

We first investigated the effect of dataset size on segmentation accuracy. We divided the 270 patients in the training dataset into three subgroups: dataset 1 (90 patients), dataset 2 (180 patients), and dataset 3 (270 patients). After training using each dataset, testing was performed using independent datasets (90 patients), and DSC was calculated to evaluate the optimal model. Statistical analysis was performed using Bonferroni correction to appraise the DSC among different datasets. The level of significance was set to less than 0.05.

### Experiment 2: segmentation accuracy with varying number of labels

Subsequently, we evaluated the segmentation accuracy using a varying number of the training labels. The number of patients for the training was 270. Here, we used four different ROI datasets: dataset 1 with one ROI (e.g., the prostate only), dataset 2 with two ROIs (e.g., the prostate and rectum), dataset 3 with three ROIs (e.g., the prostate, rectum, and bladder), and dataset 4 with all ROIs (the prostate, seminal vesicles, rectum, and bladder). Dataset 1 was regarded as a single-label task whereas datasets 2–4 were viewed as multi-label tasks. The output structures of all the training sets were the prostate, seminal vesicles, bladder, and rectum. After the training, testing was performed using independent datasets (90 patients), and the DSC was calculated to evaluate the optimal model. Statistical analysis was performed using Bonferroni correction to appraise the DSC among different datasets. The level of significance was set to less than 0.05.

### Experiment 3: segmentation accuracy in clinical practice

We developed an auto-segmentation model in Python and included a graphical user interface to create a stand-alone product that can run on any workstation. The segmented structures were converted to DICOM-RT files prior to their importation into an Eclipse RTPS (version 15.6; Varian Medical Systems Inc., Palo Alto, CA). The operation flow of the software includes (1) selecting a patient, (2) detecting the region of each organ, (3) confirming the outcome, and (4) exporting in DICOM-RT files. The volumetric DSC and the 95th-percentile Hausdorff distance (95%HD) between the predicted and manual segmentation were calculated to assess the performance of the segment accuracy.

## Results

### Computation time

The time required to create a model was 10 h, and the average and maximum complete segmentation time was 0.12 s per slice and 0.20 s per slice, respectively. The computer used in this study had an Intel Core Xeon CPU, single NVIDIA Quadro P600 GPU, and 32 GB RAM.

### Experiment 1: segmentation accuracy using datasets of different sizes

**Fig. 2:**
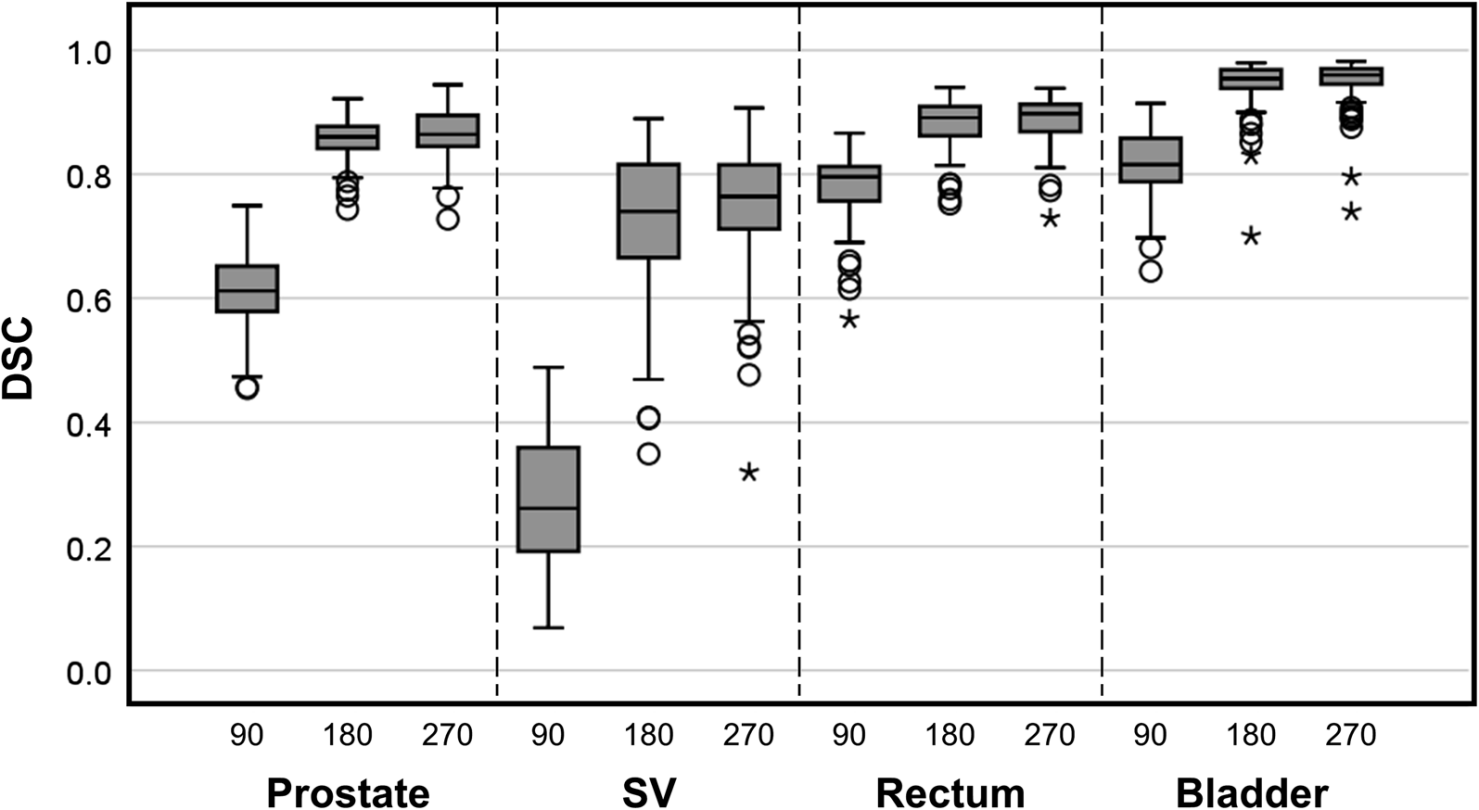
Boxplot of dice similarity coefficients with increasing dataset size. The horizontal axis shows the dataset size for each ROI

**Fig. 3:**
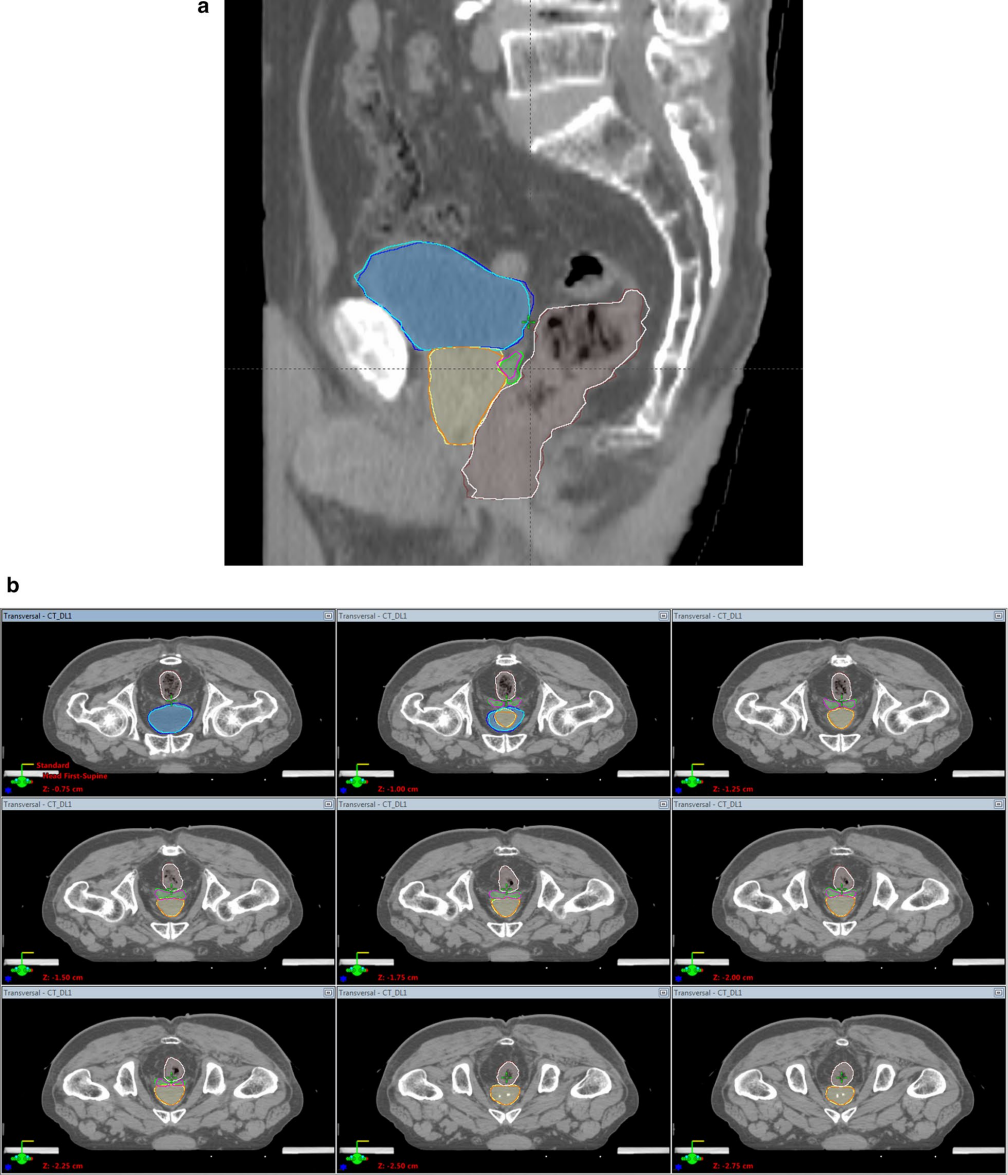
Examples of segmentation of the **a** sagittal and **b** multiple axial planes when building a model using data from 270 patients. The blue, yellow, green, and brown contours are the predicted boundaries of the bladder, prostate, seminal vesicles, and rectum, respectively. The others are the ground truth boundaries identified by the CNN and human experts, respectively

Figure 2 shows the DSCs of the training datasets as the number of the training data increased. The DSCs are reported only for the testing datasets. The median (interquartile range) DSCs for dataset 3 (270 patients) were 0.86 (0.85–0.89), 0.76 (0.71–0.81), 0.90 (0.87–0.91), and 0.96 (0.95–0.97) for the prostate, seminal vesicles, rectum, and bladder, respectively. For the segmentation accuracy of the prostate, the median DSC were 0.61 for dataset 1 (90 patients), 0.86 for dataset 2 (180 patients), and 0.86 for dataset 3 (270 patients), respectively. The median DSC for each ROI increased significantly as the number of training data increased from 90 to 180 (*p* < 0.05). When additional 90 cases were considered, the median DSCs became slightly higher (approximately 0.02 points for all ROIs); nonetheless, the differences were insignificant. The predicted segmentation of a representative patient is shown in Fig. 3. All the ROIs evidenced acceptable segmentation accuracy.

### Experiment 2: segmentation accuracy with a varying number of training labels

**Fig. 4:**
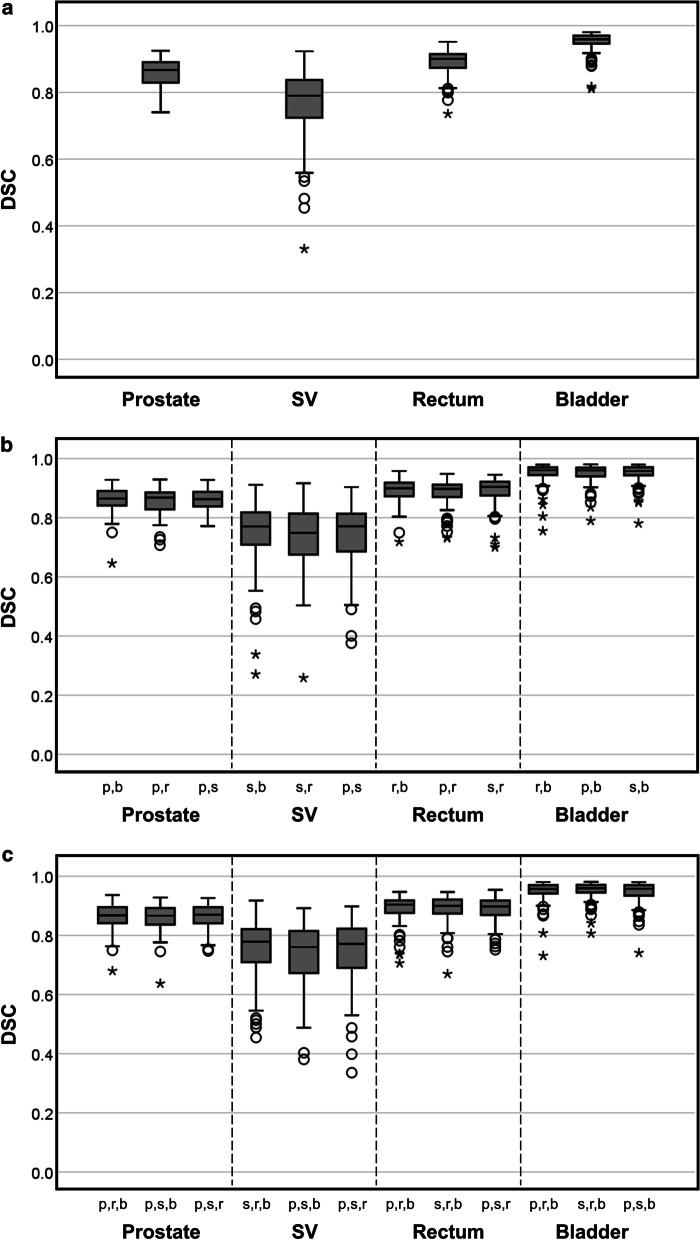
Boxplot of the dice similarity coefficients of the testing dataset using different training methods: models trained using **a** one ROI, **b** two ROIs, and **c** three ROIs. P, B, R, and S on the horizontal axis denote the prostate, bladder, rectum, and seminal vesicles, respectively

The DSCs obtained with a varying number of labels are shown in Fig. 4. The median (interquartile range) DSCs for the prostate, seminal vesicles, rectum, and bladder for all the training methods were 0.87 (0.84–0.89), 0.77 (0.69–0.82), 0.90 (0.87–0.92), and 0.96 (0.94–0.97), respectively. No significant differences were observed when one-, two-, three-, and four-ROIs training datasets were used. All the DSC quartile variations were < 5% regardless of the training method employed. Furthermore, the DSC distributions were similar for all the models; therefore, the segmentation did not depend on the type of training model used or the labeling.

### Experiment 3: segmentation accuracy in clinical practice

Based on the results in Experiments 1 and 2, the prediction model with 270 patients and four ROIs were used as optimal models to assess the segmentation accuracy. The median (interquartile range) DSC values for the prostate, seminal vesicles, rectum, and bladder were 0.82 (0.79–0.84), 0.71 (0.67–0.77), 0.89 (0.86–0.91) and 0.95 (0.94–0.96), respectively. The median (interquartile range) of 95%HD value was shown in 3.23 mm (2.99–3.42 mm) for the prostate, 3.82 mm (3.49–4.29 mm) for the seminal vesicle, 2.65 mm (2.39–2.92 mm) for the rectum, and 4.18 mm (3.52–4.77 mm) for the bladder, respectively.

## Discussion

The auto-segmentation of the prostate and surrounding ROIs effectively reduced inter-observer variation and the time required for the segmentation prior to radiotherapy [8–14]. Empirically, this is the first study developing a fully residual deep CNN-based auto-segmentation method for Asians by implementing in-house software. This can be used on the commercial RTPS in clinical practice to assess the auto-segmentation accuracy in the male pelvic region.

As expected, the segmentation accuracy improved significantly when the training image datasets from 180 (rather than 90) patients were used. The DSCs of all the ROIs created using the CT data on 180 patients were comparable to those of previous reports [9–14]. The segmentation accuracies obtained by using data from 270 (rather than 180) patients for training did not improve slightly. There are three possible explanations. First, the anatomical features of the 270 patients were similar to those of the 180 patients. Second, the manual delineation errors do not increase the DSC. Third, a fully residual deep CNN is not perfect in terms of segmentation, and the accuracy varies by organ shape and size. Regarding the seminal vesicle segmentation, the DSCs improved as the training datasets increased in size; however, the values remained low because of the complex shape and small size of this ROI compared with other ROIs. This indicates that the seminal vesicle segmentation must be carefully modified in the future. A small (approximately 180 patients) prospectively labeled training dataset (which will be available in many institutions) will enable a high-performance segmentation; nevertheless, carefully segmented ROIs are required for evaluation.

We conclude that the segmentation accuracy is not affected by the number of labels used, as shown in Fig. 4. Multi-labeling affects the weights required to recognize the labels employed to segment organs during training. Additionally, weights vary when low-contrast images (for example, a smeared border between ROIs) are used for training, affecting segmentation accuracy. Therefore, we hypothesized that the segmentation accuracy revealed by the DSC would decrease on multi-labeling. However, the segmentation accuracy demonstrated by FusionNet did not change. During the fully residual deep CNN auto-segmentation, it was essential to minimize the variance between the training and testing datasets, and the anatomical features of our two datasets were similar. Moreover, we established an institutional policy for manual segmentation. This shows that the outputs of multi-label learning of the four ROIs are comparable to those of the single-label task.

Sollini et al. asserted that though the results of studies with artificial intelligence were promising, they were still inadequate in clinical settings [18]. Consequently, deliverables with artificial intelligence will be needed for accuracy in “real world [18]”. Currently, auto-segmentation including a CNN method was implemented in some commercial treatment planning support systems [19–24]. DLCExpert™ (Mirada Medical Ltd., UK), Ethos therapy system (Varian Medical Systems, Palo Alto, CA), and Limbus Contour (Limbus AI Inc., Regina, Canada) have the function of auto-segmentation using modified U-nets such as semantic segmentation [25] and BibNet [27]. The performance of this software is clinically acceptable, including segmentation accuracy and calculation time [19–24]. Nevertheless, these systems employ down and up sampling from original image resolution to prevent out-of-memory. To conduct down and up sampling, interpolation algorithm of both non-extra and extra pixel interpolation categories is generally performed. Auto-segmentation of small organs such as the seminal vesicle is difficult because of the low contrast on the pelvic CT images when interpolation is conducted. Thus, poor performance at low resolution is likely associated with loss of information within the image [28, 29]. Our method does not use down and up sampling before training the model; therefore, it is possible to conduct auto-segmentation while keeping the original image resolution.

Table 1 summarizes the methodology of auto-segmentation using deep learning reported by other studies and our study. Segmentation accuracy in our study shows comparable results in all the ROIs reported by other studies using deep learning technique [9–14, 19, 20, 24]. The accuracy of the segmentation even in a small organ such as the seminal vesicle was higher than that of other studies owing to the high input resolution images. This is one of the advantages compared to other reports; sustaining the segmentation accuracy and calculation time with the original image information.

**Table 1:** Comparison of dataset, methodology (label and network), and the similarity scores (DSC and HD) reported by other studies and our study

Author	Number of datasets (patients)			Label	Network	Evaluation metrics	ROI				Commercial application
	Training	Validation	Test				Prostate	Seminal vesicle	Rectum	Bladder	
Macomber et al. [9]	94		99	Multiple	Deep decision forests	DSC [median (IQR)]	0.75 (0.67–0.82)	0.49 (0.31–0.79)	0.71 (0.63–0.87)	0.94 (0.92–0.98)	
						HD [mm]	–	–	–	–	
Balagopal et al. [10]	136 (including tests)			Multiple	ResNeXt (3D-Unet)	DSC (mean ± SD)	0.90 ± 0.20	–	0.84 ± 0.37	0.95 ± 0.15	
						HD [mm]	–	–	–	–	
Liu et al. [11]	771	193	140	Single	Deep neural network	DSC (mean ± SD, range)	0.85 ± 0.06 (0.65–0.93)	–	–	–	
						HD [mm] (mean ± SD)	7.0 ± 3.5	–	–	–	
Zhang et al. [12]	90	10	20	Multiple	ARPM-Net	DSC (mean ± SD)	0.88 ± 0.11	–	0.86 ± 0.12	0.97 ± 0.07	
						Average HD [mm] (mean ± SD)	1.58 ± 1.77	–	3.14 ± 2.39	1.91 ± 1.29	
Wang et al. [13]	268		45	Multiple	U-net	DSC (mean ± SD)	0.89 ± 0.03	–	0.89 ± 0.04	0.94 ± 0.03	
						HD [mm]	–	–	–	–	
Kijunen et al. [14]	876		30	Multiple	3D U-net	DSC (mean)	0.82	0.72	0.84	0.93	
						HD [mm] (mean)	6.1	7.1	11.4	3.3	
Czeizler et al. [19]	87		5	Multiple	BibNet	DSC (mean ± SD)	–	–	0.75 ± 0.11	0.90 ± 0.06	
						HD [mm]	–	–	–	–	
Schreier et al. [20]	300		50	Multiple	BibNet	DSC (mean)	0.84	0.70	0.87	0.93	
						HD [mm]	–	–	–	–	
Wong et al. [24]	328		50	Multiple	U-net	DSC (minimum)	0.79	0.64	0.78	0.97	Limbus Contour
						95%HD [mm]	6.72	5.95	12.09	3.24	
Our study	270	90	90	Multiple	FusionNet	DSC [median (IQR)]	0.87 (0.85–0.89)	0.77 (0.69–0.82)	0.91 (0.87–0.92)	0.96 (0.94–0.97)	
						HD [mm]	–	–	–	–	
			20	Multiple	FusionNet	DSC [median (IQR)]	0.82 (0.79–0.84)	0.71 (0.67–0.77)	0.89 (0.86–0.91)	0.95 (0.94–0.96)	
						95%HD [mm] [median (IQR)]	3.23 (2.99–3.42)	3.82 (3.49–4.29)	2.65 (2.39–2.92)	4.18 (3.52–4.77)	

*DSC* dice similarity coefficient, *HD* Hausdorff distance, *95%HD* 95th-percentile Hausdorff distance, *IQR* interquartile range, *ROI* region of interest

The present study has several limitations, which warrant a discussion. Our model is applicable to patients in the prone position and not to those undergoing femoral head replacement. In addition, our model cannot segment the small or large bowels. Another kind of deep learning network, such as an unsupervised learning network, would enhance the performance of our model.

## Conclusion

We found that the segmentation accuracy was improved as the number of training images increased; nonetheless, the augmented data of more than 180 patients had a little gain on the segmentation accuracy. In addition, the number of labels employed was irrelevant. We also demonstrated the efficiency of the fully residual deep CNN-based segmentation model for additional prostate cancer patients.

## Supplementary Information

**Additional file 1.** Data summary in Experiments 1, 2 and 3.

## Data Availability

The dataset supporting the conclusions of this article is included in the supplementary material (Additional file 1).

## Abbreviations

CT: Computed tomography
CNN: Convolutional neural network
DSC: Dice similarity coefficient
IMRT: Intensity-modulated radiotherapy
ROI: Region of interest
RTPS: Radiation treatment planning system
VMAT: Volumetric-modulated radiotherapy
95%HD: 95Th-percentile Hausdorff distance
